# Does “all disease begin in the gut”? The gut-organ cross talk in the microbiome

**DOI:** 10.1007/s00253-024-13180-9

**Published:** 2024-05-21

**Authors:** Prem Prashant Chaudhary, Mahaldeep Kaur, Ian A. Myles

**Affiliations:** Laboratory of Clinical Immunology and Microbiology, Epithelial Therapeutics Unit, National Institute of Allergy and Infectious Disease, National Institutes of Health, Bethesda, MD 20892 USA

**Keywords:** Human microbiome, Microbial community, Human health, Bidirectional communication

## Abstract

The human microbiome, a diverse ecosystem of microorganisms within the body, plays pivotal roles in health and disease. This review explores site-specific microbiomes, their role in maintaining health, and strategies for their upkeep, focusing on oral, lung, vaginal, skin, and gut microbiota, and their systemic connections. Understanding the intricate relationships between these microbial communities is crucial for unraveling mechanisms underlying human health. Recent research highlights bidirectional communication between the gut and distant microbiome sites, influencing immune function, metabolism, and disease susceptibility. Alterations in one microbiome can impact others, emphasizing their interconnectedness and collective influence on human physiology. The therapeutic potential of gut microbiota in modulating distant microbiomes offers promising avenues for interventions targeting various disorders. Through interdisciplinary collaboration and technological advancements, we can harness the power of the microbiome to revolutionize healthcare, emphasizing microbiome-centric approaches to promote holistic well-being while identifying areas for future research.

## Introduction

**Fig. 1:**
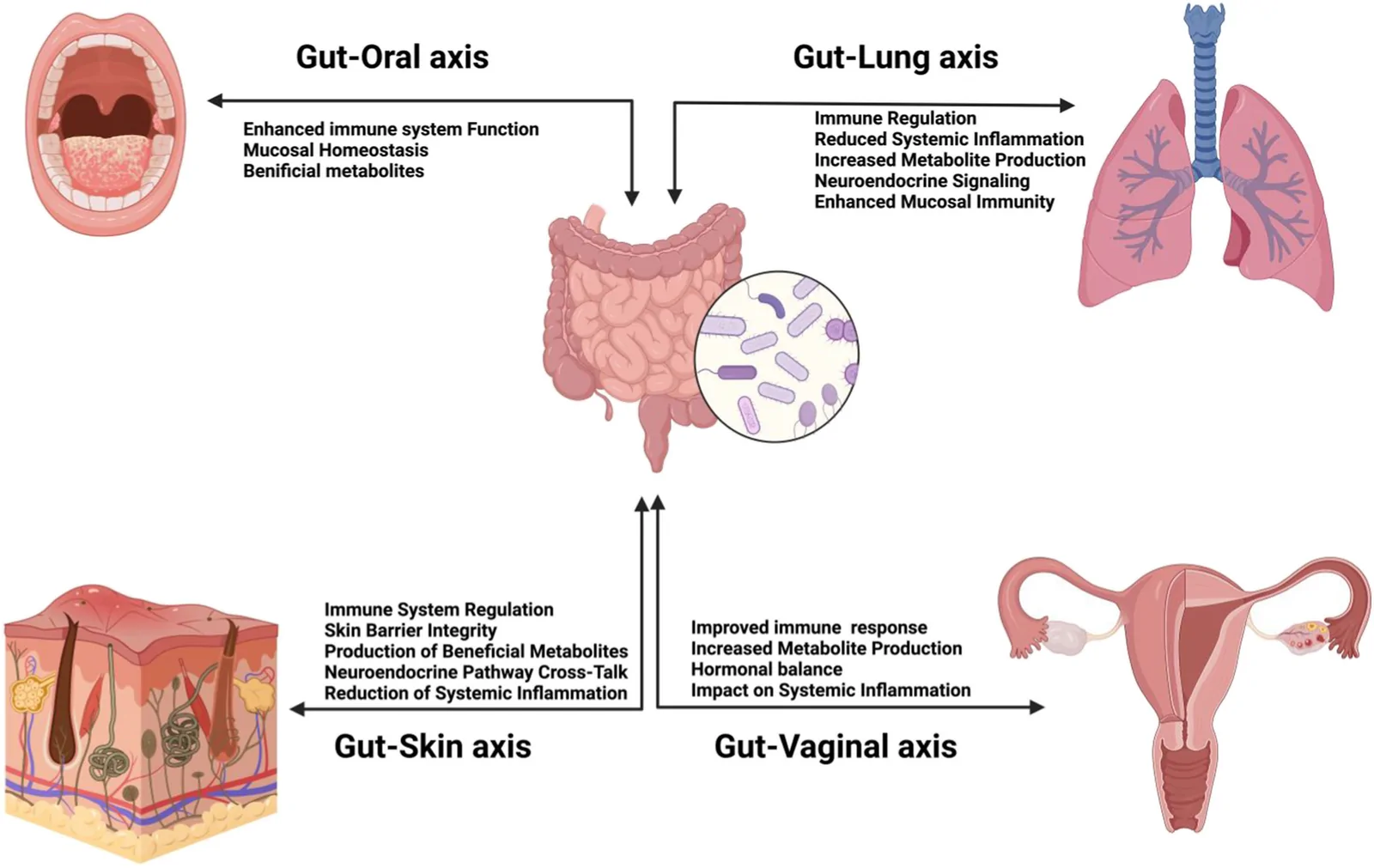
Bidirectional immunological and other alterations occurring in instances of microbial dysbiosis and a healthy microbiome across anatomical sites linked to the gastrointestinal tract

Hippocrates is thought to have said that “all disease begins in the gut.” While the human body is host to a complex and diverse community of microorganisms, collectively known as the microbiome, which inhabits various niches within the body, the gut houses the largest number of microbes (Dekaboruah et al. 2020). These microbial ecosystems play fundamental roles in maintaining health and influencing disease processes (Hou et al. 2022). Understanding the intricacies of the human microbiome and its site-specific dynamics is crucial for unraveling the mechanisms underlying human health and disease. By exploring the interconnectedness of these microbial communities and their systemic connections with the host, we aim to shed light on how alterations in one microbiome can impact others, shaping human physiology and disease susceptibility (Kho and Lal 2018). Recent research has uncovered bidirectional communication between the gut microbiota and distant microbiome sites, influencing immune function, metabolism, and disease progression (Carabotti et al. 2015; Enaud et al. 2020; Park et al. 2021; Thye et al. 2022) (Fig. 1). Moreover, emerging evidence suggests that therapeutic interventions targeting the microbiome hold promise for modulating distant microbiomes and treating a range of disorders, including oral infections, respiratory diseases, vaginal dysbiosis, and dermatological conditions (Amabebe and Anumba 2020; Hou et al. 2022; Du et al. 2022).

Through interdisciplinary collaboration and technological advancements, researchers are poised to harness the therapeutic potential of the microbiome to revolutionize healthcare (Liwinski and Elinav 2020). By developing personalized treatments that target the gut microbiome, we can aspire to improve patient outcomes and promote holistic health and well-being. This review aims to provide comprehensive understanding of the human microbiome and how the gut microbes interact with other microbes found in different parts of the body. It also points out areas where more research is needed, highlighting how focusing on the microbiome can help us understand and tackle health issues.

## Oral microbiome

The oral microbiome refers to the diverse community of microorganisms residing within the oral cavity, including bacteria, viruses, fungi, and archaea (Li et al. 2022a, 2022b). This complex ecosystem plays a pivotal role in maintaining oral health by contributing to various physiological processes such as saliva metabolism, immune modulation, and dental biofilm formation (Kilian et al. 2016). Comprising hundreds of bacterial species, the oral microbiome exists in a dynamic balance, with interactions between different microbial taxa influencing its composition and function (Dewhirst et al. 2010). While some oral bacteria are considered beneficial and contribute to oral homeostasis, others may become pathogenic under certain conditions, leading to the development of oral diseases such as periodontitis and dental caries (Lamont et al. 2018). Additionally, research has elucidated the bidirectional relationship between the oral microbiome and systemic health, with dysbiosis in the oral microbiota implicated in various systemic conditions including cardiovascular diseases, diabetes mellitus, and adverse pregnancy outcomes (Tonetti et al. 2013; Ye and Kapila 2021; Qin et al. 2022). Therefore, understanding the composition and function of the oral microbiome is essential for comprehending its role in both oral and systemic health.

## Dental health: how the microbiota affects oral hygiene

Dental health is intricately linked to the composition and balance of the oral microbiota, the diverse community of microorganisms inhabiting the oral cavity (Dewhirst et al. 2010). One key aspect of the oral microbiota influence on dental health is its role in the formation and maintenance of dental biofilms, commonly known as dental plaque. Dental plaque is a structured microbial community that adheres to tooth surfaces and is primarily composed of bacteria embedded in a matrix of extracellular polymeric substances (Marsh 2003). The microbial composition of dental plaque varies depending on factors such as oral hygiene practices, diet, and host immune responses (Marsh 2003). Certain bacterial species within dental plaque, such as *Streptococcus* mutans and *Lactobacillus* species, are known to be associated with dental caries, the most prevalent oral disease worldwide (Ahmed et al. 2014). Furthermore, the oral microbiota plays a crucial role in modulating the host immune response within the oral cavity. The interaction between oral bacteria and the host immune system can have both beneficial and detrimental effects on oral health. For instance, commensal bacteria in the oral microbiota contribute to the development and maintenance of oral mucosal immunity, protecting the host from pathogenic invaders (Yu et al. 2019). On the other hand, dysbiosis in the oral microbiota, characterized by shifts in microbial composition and function, can lead to chronic inflammatory conditions such as periodontitis, a common oral disease characterized by destruction of the tooth-supporting tissues (Genco and Borgnakke 2013).

Understanding the intricate relationship between the oral microbiota and dental health is essential for developing effective preventive and therapeutic strategies. By targeting specific components of the oral microbiota associated with oral diseases, such as caries and periodontitis, novel approaches for disease prevention and treatment can be developed. These may include interventions aimed at modulating the oral microbiota through probiotics, prebiotics, or antimicrobial agents, as well as personalized oral hygiene regimens tailored to an individual’s oral microbial profile (Marsh 2003; Haukioja 2010). Further research into the complex interactions between the oral microbiota and host factors is necessary to develop targeted interventions for maintaining oral health and preventing oral diseases.

## Oral-systemic connection: exploring the link between oral microbiome and overall health

Research has revealed intricate connections between the oral microbiome and various systemic conditions, highlighting the importance of oral health maintenance for overall well-being (Tonetti et al. 2013; Kato et al. 2018; Gao et al. 2018). One key aspect of the oral-systemic connection is the association between periodontal disease and systemic inflammatory conditions such as cardiovascular diseases, inflammatory bowel disease, and certain type of cancers (Tonetti et al. 2013; Kato et al. 2018; Zhong et al. 2021; Wang et al. 2021a, 2021b). Periodontal disease, characterized by chronic inflammation and destruction of the tooth-supporting tissues, is often driven by dysbiosis in the oral microbiota (Darveau 2010). Oral pathogens and their byproducts can enter the bloodstream through inflamed periodontal tissues, leading to systemic dissemination and contributing to the development and progression of systemic diseases (Darveau 2010).

Moreover, the oral microbiome has been implicated in the modulation of immune responses and systemic inflammation, further linking oral health to systemic health outcomes (Tonetti et al. 2013; Gao et al. 2018; Ye and Kapila 2021; Qin et al. 2022). Dysbiosis in the oral microbiota can lead to increased production of pro-inflammatory cytokines and activation of systemic immune responses, which may contribute to the pathogenesis of systemic diseases (Darveau 2010; Hajishengallis 2015). Understanding the link between the oral microbiome and systemic health is essential for developing preventive and therapeutic strategies to mitigate the risk of systemic diseases associated with oral dysbiosis. By promoting oral health and targeting dysbiotic changes in the oral microbiome, interventions aimed at reducing the risk of systemic diseases can be developed (Darveau 2010; Peng et al. 2022). In conclusion, the oral microbiome plays a crucial role in the oral-systemic connection, influencing systemic health outcomes through its involvement in periodontal disease, immune modulation, and systemic inflammation. More focused research into the mechanisms underlying the oral-systemic connection is necessary to develop effective strategies for maintaining both oral and systemic health

## Probiotic solutions: balancing the oral microbiome for improved health

Probiotics, defined as live microorganisms that confer health benefits when administered in adequate amounts, have emerged as promising agents for balancing the oral microbiome and promoting oral health (Haukioja 2010; Hill et al. 2014). These beneficial microorganisms can exert their effects by competing with and inhibiting the growth of pathogenic bacteria, modulating host immune responses, and enhancing the integrity of the oral epithelial barrier (Hill et al. 2014). Several strains of probiotic bacteria, such as *Lactobacillus* and *Bifidobacterium* species, have been investigated for their potential to improve oral health outcomes (Teughels et al. 2011). In a recent study conducted on teenagers, they received *Lactobacillus reuteri* DSM 17938/ATCC 5289 for 28 days, showing a less pronounced increase in *Streptococcus mutans* levels along with improvements in plaque, gingivitis, and bleeding (Borrell Garcia et al. 2021). Clinical studies have demonstrated that oral administration of probiotics can lead to a reduction in oral microbial counts, decreased plaque accumulation, and improvements in parameters such as gingival health and breath odor (Teughels et al. 2011; Zhang et al. 2022). Probiotics that are beneficial for oral health predominantly belong to genera such as *Lactobacillus*, *Bifidobacterium*, *Streptococcus*, and *Weissella*, alongside selected species like *Bacillus subtilis* and *Saccharomyces cerevisiae*. Notably, various strains of *Lactobacillus reuteri*, *Lactobacillus brevis*, and *Streptococcus salivarius*, among others, have been commercialized as probiotics to promote oral health. These probiotic strains are typically sourced from microorganisms isolated from the oral cavity (Allaker and Stephen 2017; Mahasneh and Mahasneh 2017).

Moreover, probiotics have been shown to have systemic effects beyond the oral cavity, including modulation of systemic immune responses and reduction of systemic inflammation (Suez et al. 2019). By promoting a balanced and healthy oral microbiome, probiotics may contribute to the prevention and management of systemic diseases associated with oral dysbiosis, such as cardiovascular diseases, diabetes mellitus, etc. (Suez et al. 2019). Overall, probiotics offer a promising approach for balancing the oral microbiome and improving oral health outcomes. Further research is needed to elucidate the specific mechanisms of action of probiotics in the oral cavity and to identify the most effective strains and formulations for oral health promotion.

## Gut connection: investigating the relationship between oral microbiome and gut health

The oral cavity which serves as a reservoir for diverse microbial populations, and disturbances in oral microbiota composition can have downstream effects on gut microbial ecology (Segata et al. 2012; Elzayat et al. 2023). Studies have shown that oral bacteria in mice can translocate to the gastrointestinal tract through swallowing, influencing the microbial composition and function of the gut microbiome (Atarashi et al. 2017). Furthermore, dysbiosis in the oral microbiome has been associated with gastrointestinal disorders such as inflammatory bowel disease (IBD), irritable bowel syndrome (IBS), and colorectal cancer (Kato et al. 2018; Zhong et al. 2021; Wang et al. 2021a, 2021b). Pathogens found in the oral cavity, along with their metabolic byproducts, have the potential to initiate systemic immune reactions and inflammation, which may worsen gut inflammation and play a role in the development of gastrointestinal diseases (Peng et al. 2022). Conversely, interventions aimed at promoting oral health, such as probiotic supplementation and improved oral hygiene practices, may have beneficial effects on gut health. Probiotics have been shown to modulate gut microbial composition and improve gastrointestinal symptoms in certain conditions (Allaker and Stephen 2017; Mahasneh and Mahasneh 2017; Suez et al. 2019).

Smoking also significantly influences the oral microbiome, resulting in dysbiosis characterized by decreased microbial diversity and increased abundance of pathogenic species. This imbalance is associated with various oral health issues, including periodontal disease, dental caries, and oral cancer. Studies have shown that smoking promotes the growth of harmful bacteria such as *Porphyromonas gingivalis* and *Tannerella forsythia*, while reducing beneficial species like *Streptococcus salivarius* and *Lactobacillus* species. Consequently, smoking disrupts the natural balance of the oral microbial ecosystem (Wu et al. 2016; Nagakubo and Kaibori 2023).

## Lung microbiome

The lung microbiome once thought to be sterile is now recognized as a diverse microbial community residing within the lower respiratory tract. Comprising bacteria, fungi, viruses, and archaea, the lung microbiome plays a crucial role in maintaining respiratory health and homeostasis (Dickson et al. 2013). Dysbiosis in the lung microbiome has been associated with respiratory diseases such as asthma, chronic obstructive pulmonary disease (COPD), idiopathic pulmonary fibrosis (IPF), and cystic fibrosis (Erb-Downward et al. 2011; Dickson et al. 2013; Garcia-Nunez et al. 2014; Campbell et al. 2023). Furthermore, the lung microbiome interacts with the host immune system, modulating immune responses and inflammation within the respiratory tract. Dysregulated immune-microbial interactions can contribute to the pathogenesis of respiratory diseases and exacerbate disease severity (Huffnagle et al. 2017).

Understanding the composition and function of the lung microbiome is essential for elucidating its role in respiratory health and disease. Targeted interventions aimed at modulating the lung microbiome, such as probiotics, antimicrobial agents, and microbiota transplantation, hold promise for improving respiratory outcomes and managing lung diseases (Cookson et al. 2018; Li et al. 2022a, 2022b; Yuksel et al. 2023). Overall, the lung microbiome is a dynamic microbial ecosystem that influences respiratory health through interactions with the host immune system. More comprehensive research into the composition, function, and impact of lung microbiome on respiratory diseases is necessary to develop novel therapeutic strategies and improve patient outcomes.

## Respiratory health: unraveling the importance of the lung microbiome

Exploring the lung microbiome unveils its crucial role in respiratory health. Recent advancements in sequencing technologies have enabled the identification of diverse microbial communities inhabiting the lower respiratory tract collectively called as lung microbiome. While the lung microbiome in healthy individuals is characterized by low microbial biomass and high diversity, dysbiosis in this ecosystem has been linked to various respiratory diseases, including asthma, chronic obstructive pulmonary disease (COPD), cystic fibrosis and many more (Erb-Downward et al. 2011; Dickson et al. 2013; Garcia-Nunez et al. 2014; Campbell et al. 2023).

Several key bacterial species have been identified as important contributors to respiratory health. For instance, studies have shown that the presence of commensal bacteria such as *Streptococcus*, *Prevotella*, and *Veillonella* in the lung microbiome is associated with improved lung function and reduced risk of respiratory infections (Segal et al. 2016). In contrast, dysbiosis of microbes is characterized by an overabundance of pathogenic bacteria like *Haemophilus influenzae*, *Moraxella catarrhalis*, and *Pseudomonas aeruginosa* that have been linked to increased inflammation, exacerbation of respiratory symptoms, and disease progression in mouse models (Boutin et al. 2017; Versi et al. 2023). Studies comparing respiratory microbiomes in BALF (bronchoalveolar lavage fluid), and sputum of COPD patients and healthy subjects found increased relative abundance of *Moraxella*, *Streptococcus*, *Proteobacteria*, *Veillonella*, *Eubacterium*, and *Prevotella* sp. in COPD (Ghebre et al. 2018; Haldar et al. 2020; Li et al. 2024).

Another study by Dickson et al. (2017) demonstrated that patients with COPD exhibited a shift in lung microbiome composition compared to healthy controls, with an increase in *Proteobacteria* and a decrease in *Firmicutes*. This profile of dysbiosis was associated with increased airway inflammation and exacerbation frequency, highlighting the role of the lung microbiome in disease pathogenesis. Similarly, in cystic fibrosis, dysbiosis characterized by the dominance of opportunistic pathogens such as *Pseudomonas aeruginosa*, has been linked to progressive lung function decline and poor clinical outcomes (Twomey et al. 2013; Versi et al. 2023). Along with bacterial communities, fungi also have its role to play, as *Aspergillus* showed the greatest distinction between healthy subjects and individuals with non-CF bronchiectasis, with its prevalence correlating with exacerbation events (Mac Aogain et al. 2018; Li et al. 2024).

## Airway diseases: addressing microbial imbalances in respiratory conditions

A study highlighted the association between dysbiosis in the lung microbiome and increased pulmonary inflammation, emphasizing the need to target microbial imbalances for managing respiratory conditions (Segal et al. 2016). Additionally, Dickson et al. (2017) identified specific bacterial topography within the healthy human lower respiratory tract, laying the groundwork for understanding microbial communities in respiratory health and disease. Huang et al. (2015) demonstrated correlations between the airway microbiome composition and disease severity, underlining the potential role of microbial imbalances in exacerbating asthma symptoms. Similarly, in COPD dysbiosis characterized by an overabundance of pathogenic bacteria associated with increased inflammation and exacerbation frequency, suggesting microbial interventions as potential therapeutic targets (Segal et al. 2016). Another study conducted in patients with bronchiectasis demonstrated that *P. aeruginosa* was independently associated with increased mortality in patients with frequent exacerbations (Araujo et al. 2018). So, managing this specific microbe could be useful in these specific patient groups.

## Therapeutic approaches: strategies for enhancing lung microbiome for respiratory well-being

Therapeutic approaches aimed at enhancing the lung microbiome for respiratory well-being are promising avenues for managing airway diseases. Studies have explored diverse strategies to modulate the lung microbiome, including probiotic supplementation, antimicrobial therapy, and microbiota transplantation (Mortaz et al. 2013; Chmiel et al. 2014; Jang et al. 2020; Wu et al. 2021; Du et al. 2022). For example, Yeo et al. (2014) demonstrated the potential of probiotics containing *Lactobacillus fermentum* CJL-112 strain to prevent influenza virus infection by activating the T-helper cells. In vitro studies have shown that *L. rhamnosus* NutRes1 possesses the ability to diminish inflammatory mediators produced by smoking-activated human macrophages (Mortaz et al. 2015). Another research conducted on mice models suggests that oral administration of *L. paracasei* NCC2461 could offer effective protection for female BALB/c mice with asthma (Pellaton et al. 2012; Natalini et al. 2023). Studies also highlighted the importance of targeted antimicrobial therapy in restoring microbial homeostasis and alleviating respiratory symptoms (Pellaton et al. 2012; Natalini et al. 2023).

In a study conducted on adults with idiopathic pulmonary fibrosis, adding co-trimoxazole or doxycycline to standard care did not improve outcomes (Martinez et al. 2021). Antimicrobial therapy also failed to enhance survival or slow lung function deterioration (Chen et al. 2021a, 2021b). In a study on mice, fecal microbiota transplantation coupled with high fiber diet mitigates the progression of emphysema by reducing inflammation and apoptosis. During microbiome analysis it has been seen that bacterial families like *Bacteroidaceae* and *Lachnospiraceae* increased after both fecal microbiota transplantation (FMT) and high-fat diet (HFD). These families are known for breaking down fiber into short-chain fatty acids (SCFAs) which may indirectly affect lung health gut-lung axis by modulating systemic inflammation and immune function mediated through SCFA production (Jang et al. 2020). Integrative approaches combining microbiome analysis with host genetics and environmental factors may provide deeper insights into microbial dysbiosis and its impact on respiratory conditions (Cribbs et al. 2016; Singh et al. 2022). With continued advancements in high-throughput sequencing technologies and computational tools, researchers can unravel the complexities of the lung microbiome with unprecedented resolution (Segal et al. 2016; Clarke et al. 2018). Integrating multi-omics approaches and longitudinal studies will provide comprehensive insights into the dynamic nature of the lung microbiome and its role in respiratory diseases (Singh et al. 2022). Ultimately, targeted interventions aimed at modulating the lung microbiome may pave the way for personalized therapeutics and improved outcomes for individuals with respiratory conditions.

## Understanding the influence of lung microbiome on gut health (lung-gut axis)

Recent studies have highlighted bidirectional communication pathways between these systems, shedding light on how alterations in one microbiome can affect the other (Madan et al. 2012; Marsland et al. 2015; Dickson et al. 2016; Budden et al. 2017). While the gut and respiratory tract microbiota share similar phylum structures, their dominant bacterial members differ. *Actinobacteria*, *Firmicutes*, and *Bacteroidetes* prevail in the gut, whereas *Proteobacteria*, *Firmicutes*, and *Bacteroidetes* dominate in the lungs (Trivedi and Barve 2020). Recently, Qu et al. (2022) suggested that gut-lung axis plays a significant role in COPD, as the cross talk between the microbial species at both sites are impacting lung health and immunity.

In another study conducted on pediatric cohorts, reduction in the population of *A. muciniphila* and *F. prausnitzii* has been associated with an increased risk of allergic asthma in patients. Authors also concluded that possibly *F. prausnitzii* and *A. muciniphila* potentially trigger the production of anti-inflammatory cytokine IL-10 while inhibiting pro-inflammatory cytokines like IL-12, indicating their potential to mitigate inflammation via secreted metabolites (Demirci et al. 2019). There is not a lot of clarity if the microbial dysbiosis and altered metabolite production in the gut initiate changes in the immune system and inflammation coupled with disease development in lungs or vice versa (Zhang et al. 2022). Moreover, therapeutic interventions targeting the gut microbiome to improve lung health outcomes have shown promise. Supplementation of *Lactobacillus* and *Streptococcus* strains at a particular dose in mice will be beneficial in reducing bacterial counts in the lungs and ultimately helps in maintaining the defense mechanism against *S. pneumoniae* (Cangemi de Gutierrez et al. 2001; Villena et al. 2006; Fukuda et al. 2011). Fukuda et al. (2011) found that specific microbial metabolites produced by gut commensals, such as short-chain fatty acids (SCFAs), could enhance gut barrier integrity and reduce susceptibility to allergic inflammation. These findings underscore the potential for modulating the gut microbiome to positively influence lung health and overall host immunity.

## Skin microbiome

The diverse microbial community on skin is vital for maintaining skin health and homeostasis. Key bacterial species such as *Staphylococcus epidermidis* and *Cutibacterium acnes* contribute to skin barrier function, immune regulation, and protection against pathogens (Fourniere et al. 2020). Numerous fungi also inhabit the skin in addition to the bacteria. The fungal species varies depending on the body part involved, for example, *Malassezia* predominantly harbors on the core body and arms, whereas *Aspergillus* spp., *Cryptococcus* spp., *Malassezia* and *Rhodotorula* spp., and *Epicoccum* spp. colonized on the foot (Skowron et al. 2021). Understanding the dynamic interplay within the skin microbiome is essential for elucidating disease mechanisms and developing targeted therapeutic interventions in dermatology. Research on the skin microbiome in various skin diseases reveals dynamic shifts in bacterial composition associated with disease pathogenesis. For instance, in eczema, a decrease in beneficial bacteria such as *Staphylococcus epidermidis* alongside an increase in potentially pathogenic *Staphylococcus aureus* has been noted (Skowron et al. 2021). Psoriasis is characterized by alterations in the skin microbiome, including an increase in *Streptococcus* spp. and reduced bacterial diversity (Celoria et al. 2023). *Acne vulgaris* is often linked with the colonization of *Propionibacterium acnes* in hair follicles, and contributes to an inflammatory acne lesion. For the microbiome associated with rosacea, studies suggest potential increases in bacteria like *Bacillus oleronius* (McLaughlin et al. 2019). These findings underscore the importance of understanding microbial dynamics in skin diseases for developing targeted therapeutic interventions aimed at restoring microbial balance and ameliorating disease symptoms.

## Dermatological insights: understanding the microbial impact on skin conditions

Dermatological research has increasingly emphasized the pivotal role of the skin microbiome in the pathogenesis of various skin conditions, providing valuable insights into disease mechanisms and potential therapeutic strategies. Eczema, characterized by compromised skin barrier function and inflammation, demonstrates microbial dysbiosis with a reduction in beneficial *Staphylococcus epidermidis* and an elevation in pathogenic *Staphylococcus aureus* (Pessoa et al. 2023; Chaudhary et al. 2023a, 2023b)*.* Similarly, psoriasis, a chronic inflammatory disorder, is associated with alterations in the skin microbiome, including an overabundance of *Streptococcus* spp. and diminished bacterial diversity (Celoria et al. 2023). *Acne vulgaris*, one of the most prevalent dermatological conditions globally, involves the colonization of *Propionibacterium acnes* within pilosebaceous units, contributing to the formation of inflammatory lesions (Vasam et al. 2023). Furthermore, there is emerging evidence implicating the skin microbiome in rosacea, with potential increases in bacteria such as *Bacillus oleronius* (Daou et al. 2021). Understanding these microbial dynamics not only elucidates disease pathogenesis but also offers avenues for targeted therapeutic interventions aimed at restoring microbial balance and ameliorating disease symptoms, thereby paving the way for personalized dermatological treatments (Byrd et al. 2018; Myles et al. 2018).

## Topical treatments: utilizing microbiome for skin health enhancement

Clinical investigations have underscored the efficacy of microbiome-targeted interventions in rebalancing microbial communities, ameliorating inflammation, and fortifying the skin barrier. For instance, formulations enriched with probiotics or prebiotics have demonstrated effectiveness in promoting the growth of beneficial skin bacteria, such as *Staphylococcus epidermidis*, while concurrently suppressing the proliferation of pathogenic species like *Staphylococcus aureus* (Di Lodovico et al. 2020). Additionally, postbiotics, including metabolic byproducts of probiotic bacteria, exhibit potent anti-inflammatory and antimicrobial properties, contributing to overall skin health enhancement (Prajapati et al. 2023). Furthermore, emerging therapeutic modalities such as bacteriophage therapy hold promise in selectively targeting and eradicating pathogenic bacteria in the skin microbiome, providing novel avenues for managing skin disorders. In addition, previous studies also reported that Gram negative commensal bacteria *Roseomonas mucosa* isolated from healthy individuals improved the outcomes of atopic dermatitis in cell culture and mouse models by inhibiting the growth of *S. aureus* (Myles et al. 2020; Zeldin et al. 2023; Barbian et al. 2023).

Moreover, examples from traditional practices, such as the use of fermented skincare products in East Asian skincare routines, highlight the long-standing recognition of skin microbiome in maintaining skin health and beauty (Jung et al. 2014). These advancements underscore the transformative potential of microbiome-based topical treatments, ushering in a new era in dermatology where personalized, microbiome-centric approaches offer tailored solutions for optimizing skin health and well-being.

## Microbiota diversity: the crucial role in skin barrier functionality

Microbiota diversity plays a pivotal role in maintaining the functionality of the skin barrier, serving as a complex ecosystem that interacts with the host immune system and influences various physiological processes. Beneficial bacteria, such as *Staphylococcus epidermidis*, are key players in promoting skin barrier integrity. *S. epidermidis* produces antimicrobial peptides, such as bacteriocins, which inhibit the growth of pathogenic microbes and contribute to the overall balance of the skin microbiome (Severn and Horswill 2023). Another example is *Cutibacterium acnes* (formerly known as *Propionibacterium acnes*), which produces short-chain fatty acids that help regulate inflammation and lipid production, thereby supporting skin barrier function (Nakatsuji et al. 2017; Almoughrabie et al. 2023).

Conversely, dysbiosis characterized by a decrease in microbial diversity, particularly in beneficial species, can compromise the skin barrier. Reductions in diversity are associated with an overgrowth of pathogenic bacteria like *Staphylococcus aureus*, which can disrupt the balance of the skin microbiome and contribute to skin barrier dysfunction (Myles et al. 2016). In conditions such as eczema, where the skin barrier is impaired, there is often a decrease in beneficial bacteria like *S. epidermidis* and an increase in potentially pathogenic species (Kong et al. 2012). Furthermore, alterations in microbial diversity have been observed in various skin disorders, including psoriasis. Studies have shown reduced bacterial diversity in psoriatic skin compared to healthy skin, suggesting a potential role of microbiota diversity in the pathogenesis of the disease (Chen et al. 2020).

## Gut connection: exploring how skin microbiome impacts gut health

Perturbations in the gut microbiota can exert systemic effects on the skin microbiome, impacting skin health and contributing to the pathogenesis of various dermatological conditions. For instance, dysbiosis in the gut microbiota can lead to systemic inflammation and immune dysregulation, which may manifest as skin disorders such as eczema, psoriasis, and acne (Belkaid and Segre 2014; Salem et al. 2018; Pessoa et al. 2023). Conversely, disruptions in the skin microbiome, such as alterations in microbial diversity and composition, can influence gut health through systemic inflammation and the modulation of immune responses (Mahmud et al. 2022). Prominent bacterial species implicated in this crosstalk include *Staphylococcus epidermidis* and *Cutibacterium acnes*, which play key roles in maintaining skin barrier function and immune homeostasis (Fourniere et al. 2020; Zhang et al. 2024).

The promotion of gut health through dietary interventions, probiotics, and prebiotics has emerged as a promising therapeutic approach for alleviating skin diseases (Ji et al. 2023).

Probiotics, such as *Lactobacillus* and *Bifidobacterium* species, have been shown to enhance gut barrier function, modulate immune responses, and alleviate inflammatory skin conditions like atopic dermatitis (Navarro-Lopez et al. 2019; Gao et al. 2023; Xie et al. 2023). Prebiotics, including dietary fibers and oligosaccharides, promote the growth of beneficial gut bacteria, leading to improvements in gut microbiota composition and function (O’Callaghan and van Sinderen 2016; Fu et al. 2022; Lee et al. 2023). These interventions can mitigate systemic inflammation and restore microbial balance, thereby ameliorating skin diseases and promoting skin barrier integrity (Jung et al. 2014; Tokarek et al. 2021; Gao et al. 2023). Furthermore, emerging research highlights the role of the gut-skin axis in the pathogenesis of autoimmune skin disorders such as psoriasis and vitiligo (Navarro-Lopez et al. 2019; Thye et al. 2022). Dysregulation of the immune system in the gut can trigger inflammatory cascades that contribute to skin inflammation and tissue damage (Yoo et al. 2020; Maciel-Fiuza et al. 2023). Modulation of the gut microbiota through interventions such as fecal microbiota transplantation (FMT) and dietary modifications has shown promise in attenuating autoimmune skin diseases by restoring immune homeostasis and dampening inflammatory responses (Navarro-Lopez et al. 2019; Kim et al. 2021). By elucidating the mechanisms underlying the gut-skin axis and harnessing the therapeutic potential of microbiome-targeted interventions, clinicians can optimize patient outcomes and improve the quality of life for individuals with dermatological conditions (Baud et al. 2023).

## Vaginal microbiome

The vaginal microbiome is a complex and dynamic microecosystem that undergoes changes during the menstrual cycle as well as in the entire life of the woman. The vaginal mucosa comprised stratified squamous nonkeratinized epithelium covered by cervicovaginal secretion and it procures glucose, oxygen, and other nutrients from underlying mucosal tissues via the process of diffusion due to limited blood supply (Chen et al. 2021a, 2021b). The vagina harbors a complex consortium of microbial communities that lives in a symbiotic relationship with the host (Chaudhary et al. 2023a, 2023b). The vaginal microecosystem harbors a billion microbes in a healthy vagina and among all, *Lactobacillus* is a dominant microbe that is known to produce numerous antimicrobial compounds. A survey by Chen et al. (2021a, b) suggested that the vagina contains 10^10^–10^11^ bacteria in women of reproductive age.

## Women’s health: maintaining microbial balance in vaginal ecology

Maintaining microbial balance in vaginal ecology is crucial for health of women, as it directly influences various aspects of reproductive and overall well-being (Lehtoranta et al. 2022). The vaginal microbiota, predominantly composed of bacteria, plays a pivotal role in protecting against infections, maintaining pH balance, and supporting reproductive health (Baud et al. 2023). The healthy abundance of *Lactobacillus* species produces lactic acid to create an acidic environment hostile to pathogenic microorganisms. This acidic pH helps prevent the overgrowth of harmful bacteria, fungi, and other pathogens, reducing the risk of conditions such as bacterial vaginosis, yeast infections, and sexually transmitted infections (STIs) (Lin et al. 2021). Factors such as hormonal fluctuations, sexual activity, hygiene practices, diet, and use of antibiotics can disrupt the delicate balance of vaginal microbiota, leading to dysbiosis or an imbalance in microbial composition (Tuniyazi and Zhang 2023). Dysbiosis can result in symptoms like abnormal vaginal discharge, itching, burning, and increased susceptibility to infections.

Maintaining microbial balance in the vaginal ecosystem involves adopting practices that support the growth of beneficial bacteria while minimizing factors that promote the proliferation of harmful pathogens. One key aspect is practicing good hygiene, including gentle cleansing of the external genital area with mild, unscented soap and water. It is important to avoid douching and using scented products in the genital area, as these can disrupt the vaginal microbiota. Additionally, maintaining a healthy diet rich in fruits, vegetables, and probiotic-rich foods can support vaginal health by promoting the growth of beneficial bacteria. Probiotics, either through dietary sources or supplements, can help replenish and maintain the population of *Lactobacillus* species in the vagina that aides in the prevention of infections and maintaining a balanced microbial ecosystem (Chen et al. 2021a, 2021b; Wang et al. 2021a, 2021b).

Screening for STIs, bacterial vaginosis, and yeast infections allows for timely intervention and treatment, minimizing the risk of complications and promoting overall vaginal health (Chen et al. 2021a, 2021b). Healthcare providers may recommend targeted treatments such as antimicrobial medications or probiotic supplements specifically formulated for vaginal health to women experiencing recurrent vaginal infections or dysbiosis. These interventions aim to restore microbial balance and alleviate symptoms associated with vaginal dysbiosis (Campisciano et al. 2021).

It is also important for women to be aware of the impact of certain lifestyle choices on vaginal health. Smoking, excessive alcohol consumption, stress, and unprotected sexual activity with multiple partners can disrupt the vaginal microbiota and increase the risk of infections (Lewis et al. 2017; Froehle et al. 2021; Turpin et al. 2021). Pregnant women should receive regular prenatal care, including screenings for vaginal infections, to ensure optimal vaginal health for themselves and their babies (Baud et al. 2023).

## Reproductive health: understanding microbiota’s role in pregnancy and birth

Understanding the role of microbiota in pregnancy and birth is crucial for promoting reproductive health and ensuring positive maternal and neonatal outcomes. The human microbiota, including the vaginal, gut, and placental microbiota, undergoes significant changes throughout pregnancy, influencing various aspects of maternal health, fetal development, and birth outcomes. The vaginal microbiota plays a critical role in pregnancy by providing protection against infections and maintaining a healthy environment for fetal development (Ravel et al. 2011). During pregnancy, the composition of the vaginal microbiota may shift, with an increase in *Lactobacillus* species associated with a lower risk of adverse pregnancy outcomes such as preterm birth (Aagaard et al. 2014). The gut microbiota also plays a crucial role in pregnancy by influencing maternal immune function, nutrient metabolism, and inflammation (Collado et al. 2008). Dysbiosis of the gut microbiota during pregnancy has been associated with gestational diabetes, obesity, and other metabolic disorders (Li et al. 2021).

Furthermore, studies have highlighted the presence of a placental microbiota, challenging the traditional notion of a sterile intrauterine environment (Aagaard et al. 2014). The placental microbiota may play a role in fetal development and immune programming, although further research is needed to fully understand its impact on pregnancy outcomes. Understanding the role of microbiota in pregnancy and birth has significant implications for maternal and neonatal healthcare (Yao et al. 2021). Strategies aimed at promoting healthy microbiota during pregnancy, such as probiotic supplementation, dietary interventions, and targeted antibiotic use, may help reduce the risk of complications and improve birth outcomes (Obuchowska et al. (n.d.); Obuchowska et al. 2022). Additionally, advances in microbiome research have led to the development of novel diagnostic and therapeutic approaches for managing pregnancy-related conditions. Biomarkers derived from maternal microbiota profiles may offer valuable insights into the risk of preterm birth, preeclampsia, and other pregnancy complications, enabling early intervention and personalized care (Kumar et al. 2021).

## Probiotic interventions: strategies for harmonizing the vaginal microbiome

Probiotic interventions offer a promising strategy for harmonizing the vaginal microbiome, promoting women’s health, and preventing or treating various vaginal dysbiosis-related conditions. Probiotics are live microorganisms that when administered in adequate amounts, confer health benefits to the host by restoring microbial balance and enhancing immune function. *Lactobacillus* species, particularly *Lactobacillus crispatus*, *Lactobacillus jensenii*, *Lactobacillus gasseri*, and *Lactobacillus iners*, are commonly found in healthy vaginal microbiota that plays a crucial role in maintaining vaginal health (Ravel et al. (n.d.)). Probiotic supplements containing these *Lactobacillus* strains (*L. crispatus* DSM32720, *L. rhamnosus* HN001, *L. crispatus* LMG S-29995, *L. acidophilus* GLA-14 and *L. crispatus* DSM32717) have been shown to effectively restore and maintain a balanced vaginal microbiome by increasing the abundance of beneficial bacteria and reducing the risk of vaginal infections (Liu et al. 2023). Several clinical studies have demonstrated the efficacy of probiotic interventions in preventing recurrent bacterial vaginosis (BV) and yeast infections (Reid et al. 2001; Falagas et al. 2006). Probiotic formulations containing *Lactobacillus* strains have been shown to reduce the recurrence rate of BV and restore vaginal pH to a healthy acidic range, thereby preventing pathogenic bacteria from colonizing the vagina (Mei and Li 2022).

In addition to preventing vaginal infections, probiotics may also have therapeutic potential in managing other vaginal dysbiosis-related conditions, such as vulvovaginal candidiasis (VVC) and aerobic vaginitis (AV) (Liu et al. 2023). Probiotic strains with antimicrobial properties can compete with and inhibit the growth of pathogenic microorganisms, restoring microbial balance and alleviating symptoms associated with these conditions (Plaza-Diaz et al. 2018). Furthermore, probiotic interventions have been investigated as adjunctive therapy for the treatment of sexually transmitted infections (STIs) such as bacterial vaginosis (BV) and human papillomavirus (HPV) infection (Liu et al. 2023). By modulating the vaginal microbiome and enhancing immune function, probiotics may help reduce the risk of STI acquisition and transmission, although further research is needed to confirm their efficacy in this context (Santos et al. 2023).

When considering probiotic interventions for vaginal health, it is essential to select probiotic strains with proven efficacy and safety profiles *Lactobacillus* (*Lacticaseibacillus rhamnosus* MG4288, *Lactiplantibacillus plantarum* MG989, *Lacticaseibacillus paracasei* MG4272, *Ligilactobacillus salivarius* MG242, and *Limosilactobacillus fermentum* MG901 species are the most used probiotics for vaginal health (Park et al. 2023).

## Gut connection: exploring the influence of vaginal microbiome on gut health

The relationship between the vaginal microbiome and gut health is an emerging area of research that highlights the interconnectedness of these two microbial ecosystems and their impact on overall health (Mestrovic et al. 2020). Studies have shown that alterations in the vaginal microbiome can influence gut health and vice versa. For example, changes in vaginal microbial composition, such as a decrease in *Lactobacillus* species and an increase in pathogenic bacteria, have been associated with gastrointestinal (GI) disorders such as irritable bowel syndrome (IBS) and inflammatory bowel disease (IBD) (Yeoh et al. 2021; Bar et al. 2023). Conversely, disturbances in composition of gut microbiota, often induced by factors like diet, stress, antibiotics, and infections, can affect vaginal health by disrupting hormonal balance, immune function, and microbial diversity. Dysbiosis of the gut microbiota has been linked to an increased risk of vaginal infections, including BV and VVC (Tuddenham et al. 2019; Han et al. 2021). Furthermore, emerging evidence suggests that the transmission of microbial communities between the gut and vagina may occur through various routes, including the bloodstream, mucosal surfaces, and shared anatomical pathways (Takada et al. 2023) .

Probiotic supplementation is one promising approach for modulating both vaginal and gut microbiota composition and promoting overall health. *Lactobacillus crispatus* and *Lactobacillus rhamnosus* BMX 54 are the commonly used strains in probiotic formulations for vaginal health and confer benefits to gut health by enhancing immune function, reducing inflammation, and inhibiting the growth of pathogenic bacteria (Recine et al. 2016; Kyser et al. 2023). Similarly, probiotics containing beneficial bacteria such as *Bifidobacterium* and *Lactobacillus* species have demonstrated efficacy in restoring vaginal microbial balance and preventing or treating vaginal infections. By promoting a balanced microbiome in both the gut and vagina, probiotic interventions may contribute to improved overall health outcomes. Understanding the influence of the vaginal microbiome on gut health and vice versa opens new avenues for research and therapeutic interventions aimed at promoting holistic approaches to health and wellness.

## Gut microbiome

The gut microbiome housing a diverse spectrum of microorganisms thriving within the gastrointestinal tract forms a dynamic ecosystem vital for human health (Qin et al. 2012; Thursby and Juge 2017). Influenced by a plethora of factors such as host genetics, dietary habits, age, antibiotic administration, and environmental exposures, the equilibrium of the gut microbiome is intricately balanced and easily perturbed (Lynch and Pedersen 2016). Such disruptions, referred to as dysbiosis, have been implicated in the onset of numerous diseases, spanning inflammatory bowel disorders, metabolic irregularities, autoimmune afflictions, and even neurological conditions (Lynch and Pedersen 2016; Marchesi et al. 2016). The connection between different body regions and diseases stemming from microbial imbalance is explored, identifying particular microbial groups showing excess and deficiency. Furthermore, it discusses the use of probiotic treatments to address these specific conditions (Table 1).

**Table 1:** Correlation between various body sites and the diseases resulting from microbial dysbiosis, delineating the specific microbial taxa exhibiting overabundance and depletion. Additionally, it outlines the probiotic interventions employed for the management of respective conditions

Body site	Disease associated with body site	Over-abundant microbes	Under-abundant microbes	Potential probiotic treatments
Gut	Irritable bowel syndrome (IBS)	Firmicutes	Bacteroidetes	*Lactobacillus plantarum*
	Crohn’s disease	*Escherichia coli*	*Faecalibacterium prausnitzii*	*Bifidobacterium infantis* DSM20088/ATCC15697
	Ulcerative colitis	*Fusobacterium nucleatum*	*Akkermansia muciniphila*	*Lactobacillus casei*
	Celiac disease	*Escherichia coli*	*Bifidobacterium bifidum*	Lactobacillus acidophilus
	Diverticulitis	*Streptococcus spp.*	*Ruminococcus spp.*	*Lactobacillus paracasei* *B21060* and *F19*
	Small intestinal bacterial overgrowth	*Klebsiella pneumoniae*	*Lactobacillus spp.*	*Saccharomyces boulardii*
	Alcoholic liver disease	*Enterococcus faecalis*	*Akkermansia muciniphila*	*Lactobacillus acidophilus*, *Lactobacillus fermentum*, and *L. rhamnosus* GG
	Non-alcoholic fatty liver disease (NAFLD)	*Klebsiella pneumoniae*	*Bacteroides fragilis*	*Bifidobacterium animalis ssp.* lactis MB 2409 (DSM 23733), *Bifidobacterium MB 109* (DSM 23731), and *Bifidobacterium longum ssp. longum* BL04 (DSM 23233)
	Obesity	*Enterobacter cloacae*	*Akkermansia muciniphila*	*Bifidobacterium infantis*, *Bifidobacterium breve*, *Bifidobacterium longum*, *Streptococcus thermophilus*, *Lactobacillus paracasei*, *Lactobacillus acidophilus*, and *Lactobacillus recarurus*
	Type 2 diabetes	*Escherichia coli*	*Faecalibacterium prausnitzii*	*Bifidobacterium bifidum* YIT 10347 and *Lactobacillus johnsonii* No. 1088
	Inflammatory bowel disease (IBD)	*Fusobacterium nucleatum*	*Roseburia spp.*	*Lactobacillus rhamnosus* GG
	Gastroesophageal reflux disease (GERD)	*Enterococcus faecalis*	*Lactobacillus spp.*	*Lactobacillus casei DN-114001*, *Lactobacillus bulgaricus LB10*, and *Lactobacillus crispatus*
	Colorectal cancer	*Bacteroides fragilis*	*Faecalibacterium prausnitzii*	*Bifidobacterium longum*
	Hepatic encephalopathy	*Enterococcus faecalis*	*Bacteroides fragilis*	*Lactobacillus rhamnosus*
	Pancreatitis	*Klebsiella pneumoniae*	*Lactobacillus spp.*	*Saccharomyces boulardii*
	Gallstones	*Enterococcus faecalis*	*Bacteroides fragilis*	*Lactobacillus*, *Bifidobacterium*, *Saccharomyces boulardii*, and *Clostridium butyricum*
	Malabsorption syndromes	*Escherichia coli*	*Bifidobacterium bifidum*	*Lactobacillus acidophilus*
	Food allergies	*Clostridium difficile*	*Bifidobacterium bifidum*	*Lactobacillus rhamnosus* GG
	Irritable bowel disease (IBD)	*Fusobacterium nucleatum*	*Ruminococcus spp.*	*Lactobacillus plantarum*
	Gastrointestinal bleeding	*Enterococcus faecalis*	*Bacteroides fragilis*	*Lactobacillus casei* and *Bifidobacterium*
Skin	Atopic dermatitis	*Staphylococcus aureus*	*Roseomonas mucosa*	*Nitrosomonas europaea* B244, *S. hominus A9*, *Mix of L. casei* (CECT9104), *B. longum ES1, B. lactis BPL1*, and *L. rhamnosus* CNCM I-4036
	Acne	*Propionibacterium acnes*	*Staphylococcus epidermidis*	*Lactobacillus acidophilus*
	Psoriasis	*Staphylococcus aureus*	*Streptococcus spp.*	*Lactobacillus pentosus* GMNL-77
	Rosacea	*Demodex folliculorum*	*Propionibacterium acnes*	*Lactobacillus paracasei* KBL382
	Eczema	*Staphylococcus aureus*	*Corynebacterium spp.*	*Lactobacillus paracasei* KBL382 s
	Wound Infections	*Pseudomonas aeruginosa*	*Staphylococcus aureus*	*Lactobacillus plantarum*, *Lactobacillus casei*, *Lactobacillus acidophilus*, and *Lactobacillus rhamnosus*
	Skin cancer	*Cutibacterium acnes*	*Staphylococcus epidermidis*	*Lactobacillus plantarum*
	Vitiligo	*Staphylococcus epidermidis*	*Malassezia spp.*	*Lactobacillus paracasei NCC2461 (ST11)*
	Hidradenitis suppurativa	*Staphylococcus aureus*	*Propionibacterium acnes*	*Lactobacillus rhamnosus*
	Diaper dermatitis	*Candida albicans*	*Staphylococcus aureus*	*Lactobacillus fermentum*
Oral	Dental caries	*Streptococcus mutans*	*Streptococcus salivarius*	*Lactobacillus reuteri*
	Oral thrush	*Candida albicans*	*Streptococcus salivarius*	*Lactobacillus acidophilus*
	Halitosis	*Porphyromonas gingivalis*	*Streptococcus salivarius*	*L. rhamnosus*
	Oral cancer	*Fusobacterium nucleatum*	*Streptococcus mitis*	*Lactobacillus plantarum*
	Oral lichen planus	*Streptococcus mutans*	*Lactobacillus spp.*	*Lactobacilli reuteri (*DSM 17938 and ATCC PTA 5289)
	Gingivitis	*Porphyromonas gingivalis*	*Streptococcus oralis*	*Streptococcus salivarius* subsp. *salivarius* strains M18 and K12, *Streptococcus oralis* subsp. *dentisani* 7746, and *Lactobacillus reuteri* ATCC PTA 5289
	Burning mouth syndrome	*Streptococcus salivarius*	*Lactobacillus salivarius*	*L. reuteri* DSM 17938 and *L. reuteri* ATCC PTA 5289
Vaginal	Bacterial vaginosis	*Gardnerella vaginalis*	*Lactobacillus spp.*	*Lactobacillus crispatus*
	Vulvovaginal candidiasis	*Candida albicans*	*Lactobacillus spp.*	*Lactobacillus acidophilus*
	Trichomoniasis	*Trichomonas vaginalis*	*Lactobacillus spp.*	*Lactobacillus reuteri*
	Atrophic vaginitis	*Gardnerella vaginalis*	*Lactobacillus spp.*	*Lactobacillus fermentum*
	Pelvic inflammatory disease (PID)	*Escherichia coli*	*Lactobacillus spp.*	*Lactobacillus iners*, *L. crispatus*, *L. gasseri*, and *L. jensenii*
	Vaginal odor	*Gardnerella vaginalis*	*Lactobacillus spp.*	*Lactobacillus rhamnosus GG*
	Vaginal yeast infection	*Candida albicans*	*Lactobacillus spp.*	*Lactobacillus acidophilus*
Lung	Cystic fibrosis	*Pseudomonas aeruginosa*	*Streptococcus spp.*	*Lactobacillus casei*, *Lactobacillus rhamnosus*, *Streptococcus thermophilus*, *Bifidobacterium breve*, *Lactobacillus acidophilus*, *Bifidobacterium infantis*, and *Lactobacillus bulgaricus*
	Asthma	*Haemophilus influenzae*	*Prevotella spp.*	*Lactobacillus casei*
	Chronic obstructive pulmonary disease	*Streptococcus pneumoniae*	*Haemophilus influenzae*	*Lactobacillus plantarum* and *Bifidobacterium*
	Lung cancer	*Fusobacterium nucleatum*	*Veillonella spp.*	*Lactobacillus plantarum* CIRM653
	Pneumonia	*Streptococcus pneumoniae*	*Staphylococcus aureus*	*Lacticaseibacillus rhamnosus CRL150513* and *Lacticaseibacillus casei CRL431*
	Tuberculosis	*Mycobacterium tuberculosis*	*Streptococcus pneumoniae*	*Lactobacillus rhamnosus*
	Acute respiratory distress syndrome (ARDS)	*Staphylococcus aureus*	*Enterococcus faecium*	*Lactobacillus reuteri*

## The impact of the gut microbiome on health and disease: insights into therapeutic strategies

Beneficial bacteria such as *Bifidobacterium* and *Lactobacillus* contribute to nutrient metabolism, synthesis of vitamins, and maintenance of intestinal barrier integrity, thus promoting digestive function (Round and Mazmanian 2009; Hill et al. 2014). Moreover, the gut microbiome interacts with the immune system, modulating immune responses and protecting against pathogens, which is essential for maintaining gastrointestinal health (Belkaid and Hand 2014). Dysbiosis of the gut microbiome, characterized by alterations in microbial composition and diversity, is associated with various digestive disorders including irritable bowel syndrome (IBS), inflammatory bowel disease (IBD), and colorectal cancer (Menees and Chey 2018; Kim and Lee 2021; Shan et al. 2022).

The microbiome of gut influence extends beyond digestion, impacting various physiological processes crucial for health maintenance. These include the regulation of metabolism, neurotransmitter production, and even immune system development (Sommer and Backhed 2013; Dicks 2022). Dysregulation of the gut microbiome has been linked to metabolic disorders like obesity and type 2 diabetes, as well as neurodevelopmental conditions such as autism spectrum disorder and depression ((Tilg and Kaser 2011; Mayer et al. 2014; Liu et al. 2020). Furthermore, disruptions in gut microbial composition have been associated with systemic inflammation, contributing to the pathogenesis of chronic diseases like cardiovascular disease and rheumatoid arthritis (Scherer et al. 2020). The emerging field of microbiome research offers insights into novel therapeutic approaches, including precision medicine strategies targeting the gut microbiota to prevent and treat a diverse range of health conditions (Lloyd-Price et al. 2016; Marchesi et al. 2016).

## Gut-brain axis: investigating the connection between gut microbiome and mood

Emerging research has highlighted the profound impact of gut microbiota on mood regulation and emotional well-being. Beneficial gut bacteria, including species like *Bifidobacterium* and *Lactobacillus*, are responsible for synthesizing neurotransmitters such as serotonin and gamma-aminobutyric acid (GABA), which are pivotal in regulating mood (Cryan and Dinan 2012; Foster and McVey Neufeld 2013). In a recent study, the efficacy of a mixture containing *Lactobacillus helveticus* R0052 and *Bifidobacterium longum* R0175 was examined, revealing favorable psychological outcomes alongside (modulating the gut microbiota, increase in short chain fatty acids and gamma-aminobutyric acid, decrease in pro-inflammatory cytokines, and increase in anti-inflammatory cytokines) decreased serum cortisol levels in human participants (De Oliveira et al. 2023). Moreover, gut microbes influence the production of neuroactive compounds, including short-chain fatty acids (SCFAs) have the ability to influence brain function and behavior (Cryan and Dinan 2012). Dysbiosis of the gut microbiome has been implicated in the pathogenesis of mood disorders such as depression and anxiety, with alterations in microbial composition and diversity observed in affected individuals particularly with decrease in microbial diversity and richness (Jiang et al. 2015; Kelly et al. 2016). Patients of depressive order have higher abundance of *Enterobacteriaceae* and *Alistipes* and decreased level of fecal bacterium abundance.

The bidirectional communication along the gut-brain axis involves intricate signaling pathways, including neural, endocrine, and immune mechanisms, that influence mood and cognitive function (Mayer and Tillisch 2011; Foster and McVey Neufeld 2013). Perturbations in the gut microbiome, induced by factors such as stress, diet, and antibiotic use, can disrupt this communication, contributing to alterations in mood and behavior (Foster and McVey Neufeld 2013; Kelly et al. 2016). Additionally, inflammation triggered by dysbiosis of the gut microbiome has been implicated in the pathophysiology of mood disorders, highlighting the role of immune activation in modulating brain function and mental health (Rook et al. 2014; Dinan and Cryan 2017). The integration of microbiome-targeted interventions, including probiotics, dietary modifications, and fecal microbiota transplantation (FMT), holds promise for restoring microbial balance and alleviating symptoms of mood disorders by targeting the gut-brain axis (Slyepchenko et al. 2017; Wallace and Milev 2017) personalized approaches to mental health care.

## Dietary strategies: promoting gut microbiome diversity for overall health benefits

Promoting gut health through dietary interventions and supplements is essential for maintaining microbial balance and supporting overall well-being. A diet rich in fiber, fruits, vegetables, and fermented foods serves as a cornerstone for gut health by providing prebiotic fibers that nourish beneficial gut bacteria (Fu et al. 2022; Bedu-Ferrari et al. 2022). Prebiotics, such as inulin, oligofructose, and resistant starch, selectively stimulate the growth of beneficial microbes like *Bifidobacteria* and *Lactobacilli*, thereby enhancing gut microbial diversity and function (Holscher 2017; Baxter et al. 2019). Probiotic-rich foods like yogurt, kefir, and kimchi contain strains of *Lactobacillus* and *Bifidobacterium* that support gut health and may alleviate gastrointestinal symptoms (Hill et al. 2014; Leeuwendaal et al. 2022).

Furthermore, dietary supplements such as prebiotics and probiotics offer convenient ways to enhance gut health (Holscher 2017; Fu et al. 2022). Moreover, synbiotic formulations, which combine prebiotics and probiotics, synergistically promote gut health by providing both substrate and live bacteria for microbial fermentation and growth (Gibson and Roberfroid 1995).

In addition to dietary interventions and supplements, lifestyle factors such as stress management, regular exercise, and adequate sleep also play crucial roles in promoting gut health. Chronic stress and sleep disturbances can disrupt gut microbial composition and function, leading to gastrointestinal symptoms and systemic inflammation (Kelly et al. 2016). Age is also an important factor which can change microbiome; elderly individuals exhibited altered abundance of 21 microbial species, indicating age-related shifts in gut and oral microbiomes in Singaporean population (Chen et al. 2022). Overall, promoting gut health through dietary interventions, supplements, and lifestyle modifications is essential for maintaining microbial balance, supporting digestive function, and enhancing overall health and well-being.

## Challenges in adopting microbiome modulation interventions

Implementing interventions to modulate the microbiome for improved systemic health and reduced inflammation faces several hurdles. Firstly, the vast complexity and diversity of the human microbiome pose challenges in developing universally applicable interventions that effectively target specific microbial imbalances. Additionally, the lack of standardized protocols for dosage, duration, and administration methods complicates consistent implementation in clinical practice. Furthermore, limited understanding of microbiome-host interactions and potential adverse effects raises safety concerns among healthcare providers. Cost and accessibility issues also restrict widespread adoption, as some interventions may be costly or unavailable to certain populations. Moreover, regulatory hurdles prolong the development and approval of novel interventions, further limiting their availability for clinical use. Addressing these challenges requires ongoing research efforts to enhance our understanding of the microbiome and develop safe, effective, and accessible interventions, alongside efforts to standardize protocols and streamline regulatory processes.

## Concluding remarks

In conclusion, this review has illuminated the individual role of site-specific microbiomes in health and diseases and intricate interplay between various microbiomes throughout the body, ranging from the oral cavity and lungs to the vagina, skin, and the central hub of the gut. Understanding these microbial communities and their connections is pivotal in unraveling the complexities of human health and disease. Through extensive research, it has become increasingly evident that the gut microbiota plays a crucial role in modulating the health of other microbiome sites and vice versa. Bidirectional communication between the gut and distant microbiomes influences physiological processes, immune responses, and susceptibility to various disorders. Moreover, the emerging field of microbiome-based therapies holds tremendous promise in revolutionizing healthcare. Harnessing the therapeutic potential of gut microbiota in the form of probiotics and fecal transplantation therapies to modulate distant microbiomes offers a novel approach for treating a myriad of diseases. From oral infections to respiratory disorders, vaginal dysbiosis, dermatological conditions, and beyond, interventions targeting the gut microbiome could pave the way for personalized, effective treatments. As we continue to delve deeper into the complexities of the microbiome and its systemic effects, interdisciplinary collaboration and innovative research methodologies will be essential. By unraveling the intricate connections between different microbiomes and leveraging the therapeutic potential of the gut microbiota, we can aspire to develop targeted interventions that promote health and combat disease across multiple body sites. This journey toward microbiome-centric healthcare holds immense promise for improving the well-being of individuals worldwide.
